# Development of a Cell-Based Functional Assay for the Detection of *Clostridium botulinum* Neurotoxin Types A and E

**DOI:** 10.1155/2013/593219

**Published:** 2013-03-07

**Authors:** Uma Basavanna, Tim Muruvanda, Eric W. Brown, Shashi K. Sharma

**Affiliations:** Division of Microbiology, Office of Regulatory Science, Center for Food Safety and Applied Nutrition, Food and Drug Administration, CPK1, HFS-712, 5100 Paint Branch Parkway, College Park, MD 20740, USA

## Abstract

The standard procedure for definitive detection of BoNT-producing *Clostridia* is a culture method combined with neurotoxin detection using a standard mouse bioassay (MBA). The mouse bioassay is highly sensitive and specific, but it is expensive and time-consuming, and there are ethical concerns due to use of laboratory animals. Cell-based assays provide an alternative to the MBA in screening for BoNT-producing *Clostridia*. Here, we describe a cell-based assay utilizing a fluorescence reporter construct expressed in a neuronal cell model to study toxin activity *in situ*. Our data indicates that the assay can detect as little as 100 pM BoNT/A activity within living cells, and the assay is currently being evaluated for the analysis of BoNT in food matrices. Among available *in vitro* assays, we believe that cell-based assays are widely applicable in high-throughput screenings and have the potential to at least reduce and refine animal assays if not replace it.

## 1. Introduction

Botulinum neurotoxins (BoNTs) are the most toxic substance known [1]. They are primarily produced by the spore-forming bacterium *Clostridium botulinum* and, in rare cases, by some strains of *Clostridium butyricum* and *Clostridium baratii* [2, 3]. Intoxication with one of the seven distinct serotypes of BoNT (A–G) causes botulism. One of 4 serotypes of BoNT (A, B, E, and F) is usually the cause of human botulism. Exposure to type A neurotoxin (BoNT/A) causes the majority of food-borne outbreaks and has been observed to cause more severe symptoms with a higher mortality [4]. BoNTs are zinc proteases that cleave and inactivate cellular proteins essential for the release of the neurotransmitter acetylcholine. BoNT/A, -C, and -E cleave the peripheral plasma membrane protein Soluble N-ethylmaleimide-sensitive factor Attachment Protein of 25 kDA (SNAP-25); BoNT/B, -D, -F, and -G cleave synaptobrevin 2, also called as vesicle-associated membrane protein-2 (VAMP-2). In addition to cleaving SNAP-25, BoNT-C also cleaves the integral plasma membrane protein, syntaxin [1]. Cleavage of these substrates inhibits neuronal exocytosis [5]. The soluble N-ethylmaleimide sensitive factor attachment protein receptor (SNARE) superfamily has become, since its discovery, the most intensively studied element of the protein machinery involved in intracellular trafficking.

Currently, the only accepted assay with which to detect active *Clostridium botulinum* neurotoxin is an *in vivo* mouse bioassay. The mouse bioassay is sensitive and robust and does not require specialized equipment. However, the mouse bioassay is slow and not practical in many settings, and it results in the death of animals. Several *in vitro* assays have been developed to detect the activities of the different BoNT serotypes [6–13]. This approach has led to Forester Resonance Energy Transfer (FRET) methods that are based on the natural substrates tagged with fluorescence dyes and their cleaved fluorescence products by BoNTs, which are then used to detect toxin activity [14–16]. Dong and coworkers described two recombinant reporters containing residues 141–206 and 33–94 of neuronal SNARE proteins SNAP-25 and synaptobrevin 2, respectively. The substrates were expressed as fusions of cyan fluorescent protein (CFP) and yellow fluorescent protein (YFP), enabling the detection of BoNT proteolysis activity by FRET. Advances in the development and optimization of FRET-based assays that detect six of seven BoNT serotypes have also been reported [17, 18]. The FRET reporters were found to be sensitive to BoNT serotypes and able to detect picomolar concentrations of the toxins in real-time assay.

The main objective of our study was to develop a cell-based assay for the detection of BoNT/A and BoNT/E, which could be automated and applied to many laboratory settings. Here, we report a cellular sensor utilizing advanced FRET-based substrates expressed as fusions of green fluorescence protein (GFP) and *Discosoma* sp. red fluorescent protein (DsRED) containing full-length neuronal SNARE protein SNAP-25. The resulting reporters are stably expressed in living cells, and the neurotoxin cleavage activity is detected either by measuring the loss of FRET or by destruction of the C-terminal fragment. These FRET-based reporters are currently being evaluated for the analysis of different food matrices and also exploring new immuno-magnetic bead separation methods to counter the food matrix interference and to increase the sensitivity of the detection. Even though the assay is thousandfold less sensitive than the mouse bioassay, cell-based assays do not require the use of animals. Further refinement of such assay will present an alternative to the mouse assay, and the ease of use could generate the confidence in field or reference laboratories capable of performing BoNT detection, leading to reduction in animal use.

## 2. Materials and Methods

### 2.1. Chemicals and Reagents

BoNT/A (Hall A strain) and BoNT/E (Alaska strain) were obtained from Metabiologics (Madison, WI). pAcGFP1-C1 and pDsRed-Monomer-C1 were purchased from Clontech Laboratories (Mountain View, CA). Full-length SNAP-25 cDNA and PC12 transplantable rat pheochromocytoma cells were purchased from ATCC (Manassas, VA). Lipofectin Transfection Reagent was obtained from Life Technologies (Grand Island, NY). Antibodies targeting SNAP-25 were purchased from Santa Cruz Biotechnology (Santa Cruz, CA). The HRP-conjugated secondary antibody was purchased from Millipore (Billerica, MA). All other reagents were obtained from Sigma-Aldrich (St. Louis, MO).

### 2.2. Generation of FRET-Based Reporters

**Figure 1:**
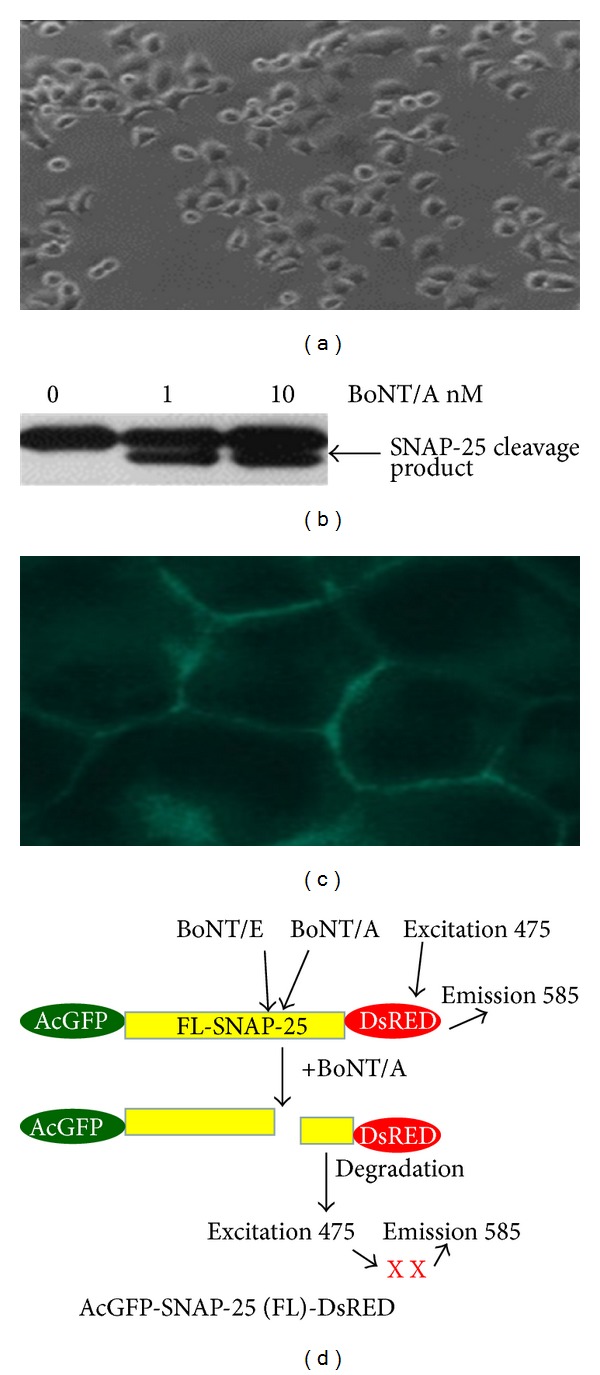
(a) Morphology of PC12 cells acquired under bright field microscopy (×40) cultured according to standard methods. (b) Sensitivity of PC-12 cell lines to BoNT/A as shown by SNAP-25 cleavage efficiency. Western blot image of PC-12 cells lysate after treatment with BoNT/A. The cleavage product marked by an arrow. (c) Immunofluorescence staining of methanol-fixed PC-12 cells showing SNAP-25 membrane localization. Image was acquired using a Nikon Eclipse Ti-E confocal microscope (Nikon Inc., USA). (d) Schematic representation of the BoNT sensor constructs. A fusion protein construct with full-length SNAP-25 as a linker between AcGFP1 and DsRED-monomer was created resulting in the AcGFP-SNAP-25-DsRed construct. Arrows indicate the site of cleavage by BoNT/A, -C, -E.

To build a neurotoxin sensor that can detect cleavage of SNAP-25 in living cells and to report toxin activity, we linked AcGFPc1 and DsRED together by using full-length SNAP-25 that can be cleaved by the appropriate BoNTs (Figure 1(d)). The construct was engineered to encode full-length SNAP-25 sequence as a linker between AcGFP1 and DsRED-Monomer using standard cloning techniques. PAcGFP-C1 was inserted in to pDsRed-Monomer-C1 by using XhoI/EcoRI restriction enzymes, resulting in AcGFP1-DsRed-Monomer construct. Full-length rat SNAP-25 cDNA was inserted in to the AcGFP1-DsRed-Monomer vector resulting in an AcGFP-SNAP-25-DsRed construct.

### 2.3. Live Cell Imaging and FRET Analysis

PC12 cells were cultured according to standard cell culture methods. A day before transfection, cells (~1 × 10^5^/mL) were seeded onto collagen-coated tissue culture dish and transfected with FRET sensor (AcGFP-SNAP-25-DsRed), donor only (AcGFPC1), and acceptor only (DsRED-monomer) cDNA constructs using a lipid-based transfection reagent according to manufacturer's instructions (Lipofectin/Life Technologies). After incubation for six hours, the transfection medium was exchanged to Dulbecco's Modified Eagle Medium (DMEM) plus 10% fetal bovine serum (FBS) to grow cells. Transfected cells were plated (~2.0 × 10^4^ cells per well) onto 4-well LabTek II chamber slides and maintained at 37°C, 5% CO_2_ in a tissue culture incubator, 24–48 hours prior to imaging. Thirty minutes prior to imaging, the culture medium was replaced with DMEM medium without FBS.

Images were acquired using a Nikon Eclipse Ti-E confocal microscope (Nikon Inc., USA) with a 1.4 numerical aperture, ×100 oil-immersion objective. Images (12 bit) of multitrack channels were with the following configuration: an argon/2 laser (25 mW, T1 and T3 = 10% of laser exposure) for the green channel (donor excitation/donor emission: Green Ex/Em), FRET channel (donor excitation/acceptor emission: FRET Ex/Em) with excitation at 488 nm, and HeNe 1 laser (T2 = 100%) for the red channel (acceptor excitation/acceptor emission: Red Ex/Em) with excitation at 543 nm. All images were acquired with exactly the same settings (4 × 4 binning, 200 msec exposure time). The background (from areas that did not contain cells) was subtracted from each raw image. PC12 cells transfected with GFP or DsRED alone were first tested to obtain the cross-talk/bleed-through values for these filter sets. Image capture and calculations were performed by using NIS-Elements software (Microsoft Corporation). For experiments involving toxin treatment, different concentrations of holotoxin (BoNT/A, trypsin-treated BoNT/E) toxin complexes were added to the cell culture media, and cells were then analyzed as described earlier.

### 2.4. Generation of Stable Cell Lines

Multiple cell lines were generated that stably express the reporter over many passages. Also, 24 h after PC12 cells were transfected with reporter constructs, growth medium was exchanged for 0.05 mg/mL G418 selection, and incubation continued until single clones appeared. Cells were transferred progressively from 24 well plates to 6 well plates as they reached confluence (1-2 weeks). Cells were ready for freezing once they were confluent on 6-well plates. When an assay was required, cells were collected and plated onto microtiter plates (NUNC, Rochester, NY) and allowed to expand for 24 h. The cells were then incubated with samples containing BoNT/A in the assay buffer (100 *μ*L of 50 nM Hepes-NaOH (pH 7.1), 5 mM NaCl, 0.1% Tween-20, 5 mM dithiothreitol (DTT), and 10 nM ZnCl_2_) and diluted and reconstituted with the culture medium for the duration of 24–72 hours to reach desired sensitivity. Cells were then washed with phosphate-buffered saline (PBS) to reduce background before collecting FRET emissions. The data was captured by measuring the total DsRED emission, converted into a ratio metric value and plotted as a function of BoNT concentration. Alternatively, FRET measurements were also taken on a Synergy 2 multimode plate reader (BioTek Instruments, Winooski, VT). For all line graphs, data shown are averages from triplicate determinations with bars indicating standard deviations.

### 2.5. Immunostaining and Blot Analysis

PC12 cells grown on a culture slide were fixed in 4% paraformaldehyde and permeabilized with 1% Triton X-100. Slides were then blocked with 0.5% BSA at RT for 2 hours and washed. Anti-SNAP-25 antibody diluted in blocking buffer (1 : 250 dilution) was added to the slides and incubated for 1 h at 4°C and washed. FITC labeled, Goat Anti-rabbit IgG antibody was used in blocking buffer and added to the plates. After slides were incubated at 37°C for 60 min, they were washed three times, and images were acquired using a Nikon confocal microscope with FITC filter.

To visualize the cleavage products of neurotoxin activity on an immune blot, PC12 cells grown on culture dishes were treated with BoNT/A (10 nM) at 37°C for 48 h. Cells were then harvested and lysed with RIPA (Radio-Immunoprecipitation Assay) buffer. The soluble protein supernatant was loaded onto a 12% polyacrylamide gel. Proteins resolved on the gel were then subjected to immunoblot analysis. Anti-SNAP-25 antibody (C-18, Santa Cruz Biotechnology) directed against the C-terminus of the protein was used to recognize the botulinum neurotoxins A and E cleavage products. Peroxidase-labeled antibodies diluted in blocking buffers were used to detect cleavage products and then visualized using a gel imager (Syngene, USA).

## 3. Results

### 3.1. Selection of a Sensitive Cell Line

We identified transplantable rat pheochromocytoma neuronal cell line (PC12 cells) (Figure 1(a)) derived from rat brain medulla for developing stable cell lines expressing FRET sensor. PC12 cells are sensitive to BoNT/A with sensitivities equivalent to primary cord neurons, as observed by the significant cleavage of SNAP-25 at BoNT/A concentration of 1 nM (Figure 1(b)). Previous reports have confirmed that the BoNT/A light chain contains a membrane localization signal and is targeted to the plasma membrane in differentiated PC12 cells [19, 20]. The immunofluorescence staining of methanol-fixed PC12 cells shows the localization of membrane-bound SNAP-25 (Figure 1(c)).

### 3.2. Design and Creation of BoNT Sensor

If the GFP and DsRED are close enough to each other, excitation of the GFP moiety will result in the sensitized emission from the DsRED moiety as a consequence of FRET. Cleavage of the linker sequence between AcGFP and DsRED separates them and abolishes FRET (Figure 1(d)). In FRET, the energy is transferred nonradioactively from donor protein to the acceptor protein when they are in very close proximity (about 50 Å) and when the emission spectrum of the donor protein overlaps with the excitation spectrum of the acceptor protein [21].

### 3.3. Generation of Stable Cell Lines and Passage Stability of the Clones

**Figure 2:**
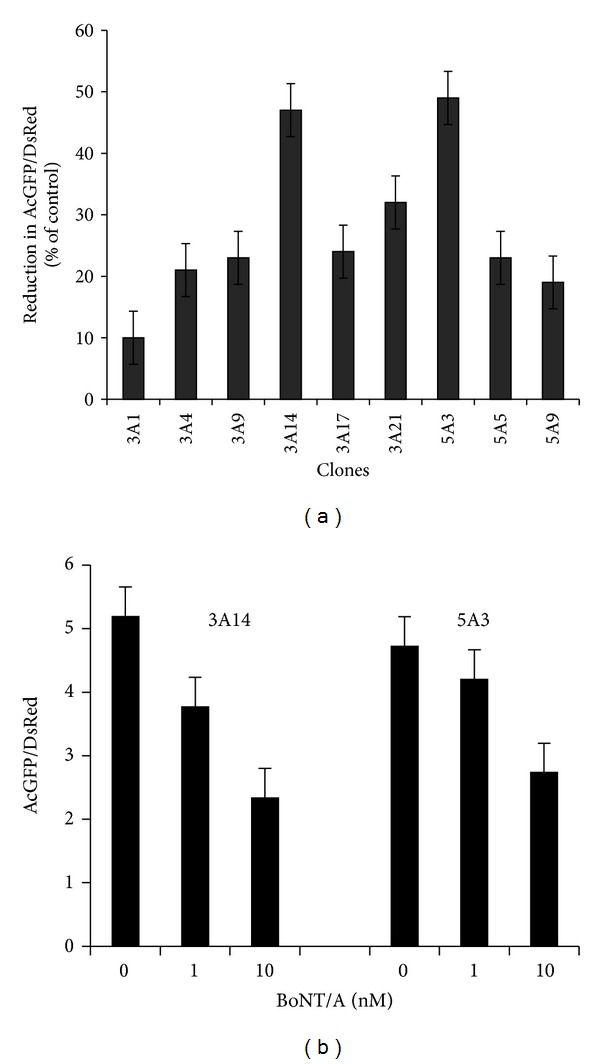
(a) Clone selection and passage stability of different clones. More than 20 clones isolated for the expression of the reporter. Clones 3A1, 3A4, 3A9, 3A14, 3A17, 3A21, 5A3, 5A5, and 5A9 were tested for the growth, performance, and stability over 20 passages and chosen for the assay development. Clones were frozen at each passage, and cells were thawed and grown for an additional passage before being subjected to the sensor assay protocol with 20 nM BoNT/A treatment. The data shown here is for passage 15. Emission ratios were calculated by dividing the emission at 585 nm (DsRED) by the emission at 475 nm (GFP) ratio compared to control experiments (no BoNT treatment). Data are presented as means ± SD (*n* = 3). (b) Two clones, 3A14 and 5A3, chosen for further subcloning and FRET studies due to their superior performance and stability for more than 20 passages and subjected to 0–10 nM BoNT/A. Emission ratios were calculated by dividing the emission at 585 nm (DsRED) by the emission at 475 nm (GFP). Data are presented as means ± SD (*n* = 3).

We generated stable cell lines that stably express the reporter protein over many passages, and the data from the P15 for nine clones are shown in Figure 2(a). Out of nine subclones (3A1, 3A4, 3A9, 3A14, 3A17, 3A21, 5A3, 5A5, and 5A9) tested for stability, only two subclones, 3A14 and 5A3, constitutively expressed AcGFPC1-SNAP-25-DsRed reporter and responsive to BoNT/A at 1 and 10 nM (Figure 2(b)). Subclones 3A14 and 5A3 were chosen for FRET studies.

## 4. Discussion

### 4.1. Monitoring BoNT/A and BoNT/E Holotoxin Activity in Living Cells

**Figure 3:**
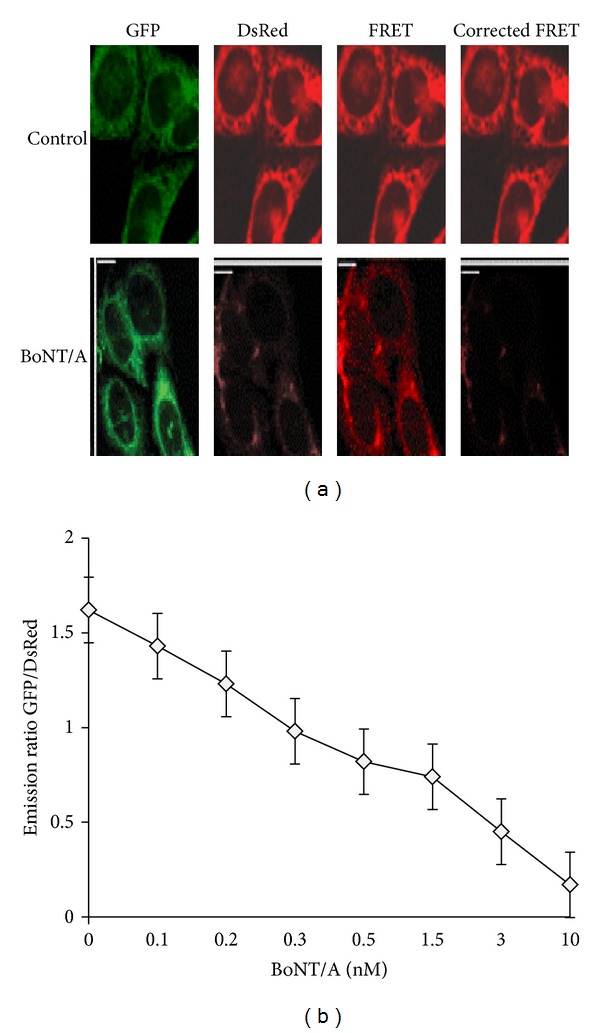
(a) Measuring toxin sensor in living cells. The stable clone 5A3 expressing the AcGFP-SNAP-25-DsRED sensor was treated with 10 nM BoNT/A holotoxin, and the FRET signals of 100 cells were analyzed after 72 hours. (as described in Materials and Methods). Control cells not treated with toxins were analyzed in parallel (upper panel). Images of representative cells are shown. This sensor yielded significant FRET (upper “corrected FRET”), which was abolished after cells were treated with BoNT/A (72 h, lower “corrected FRET”). Three images (GFP, FRET, and DsRED) were taken for each set of cells sequentially, using exactly the same settings. FRET signal was measured by exciting AcGFP and detecting DsRED signal. (b) Measurements of C-terminal reporter fragment degradation in clone 5A3 using a microplate reader (BioTek). The GFP and DsRED emissions were collected by fluorescence microplate reader (BioTek). Emissions were plotted as a function of BoNT/A at 0.03, 0.05, 0.15, 0.3, 1.5, 3.0, and 10 nM concentrations. Data are presented as means ± SD (*n* = 3).

**Figure 4:**
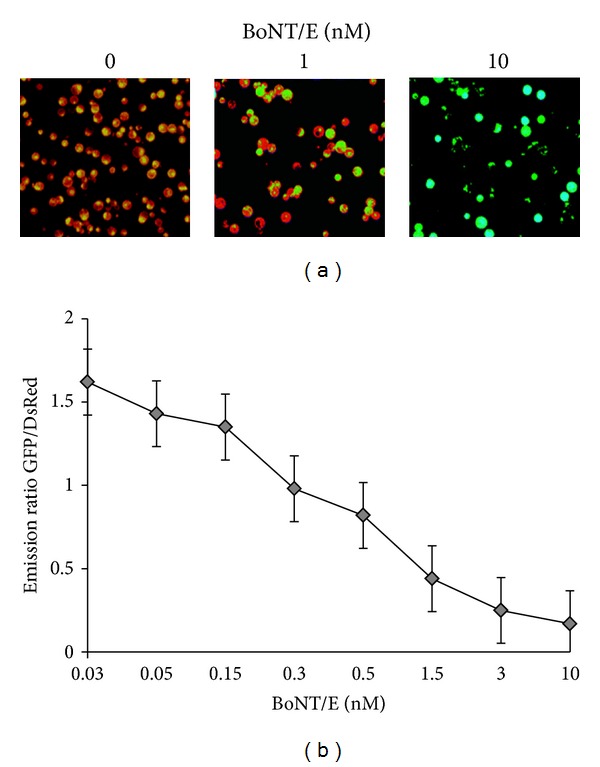
(a) BoNT/E responses of the clone 3A14. PC12 cells were grown in 96 well plates treated with the indicated BoNT/E concentration for 72 h and imaged using fluorescence microscopy. Pseudocolored images indicate the distribution of the GFP/DsRED fluorescence ratio within the cells. (b) Monitoring BoNT/E activity using a microplate reader. The GFP and DsRED emissions were also collected by fluorescence microplate reader (BioTek). Emissions were plotted as a function of BoNT/A at 0.03, 0.05, 0.15, 0.3, 1.5, 3.0, and 10 nM concentrations. Data are presented as means ± SD (*n* = 3).

To carry out cell-based studies, clones 3A14 and 5A3 were treated with 1–10 nM BoNT/A for 72–96 hours. The FRET signals in living cells were acquired by using three-filter set method as shown in Figure 3(a), and details are provided in the Materials and Methods. A progressive decrease in the FRET ratio was observed over time. This sensor yielded significant FRET (upper “corrected FRET”), which was abolished after cells were treated with BoNT/A for 72 h (lower “corrected FRET”). We also tested the cleavage activity of BoNT/E on these clones (3A14 and 5A3), which also resulted in the abolishment of FRET activity as indicated by the reduction in the red florescence in the cell population as shown in Figure 4(a). BoNT/E response of the clone 3A14 is shown in Figure 4(a), treated at 1 and 10 nM of BoNT/E concentration for 72 h, and it was captured using fluorescence microscopy. Pseudocolored images indicate the distribution of the GFP/DsRED fluorescence ratio within the cells.

### 4.2. Monitoring BoNT/A and BoNT/E Activity by Fluorescence Microplate Reader

When 5A3 clones at passage 15 were incubated with BoNT/A, the toxin is internalized resulting in the release of the BoNT/A light chain into the cytosol. The BoNT/A light chain then cleaves the reporter resulting in the release of a C-terminal reporter fragment into the cytosol that contained residues 198–206 of SNAP-25 and DsRED. The data in these studies were captured by measuring the total DsRed emission of the cells using directly excited GFP (Figure 3(b)). This will be an alternative toxin cleavage measurement in addition to FRET fluorescence measurement explained earlier in the study. Directly GFP fluorescence is also collected to normalize the cell density and fluorescence expression. Thus, DsRed emissions are divided by GFP emissions giving a ratio metric assay readout. Emissions were plotted as a function of BoNT/E concentration as shown in Figure 4(b).

### 4.3. Monitoring BoNT/A and BoNT/E Complex Activity in Living Cells

**Figure 5:**
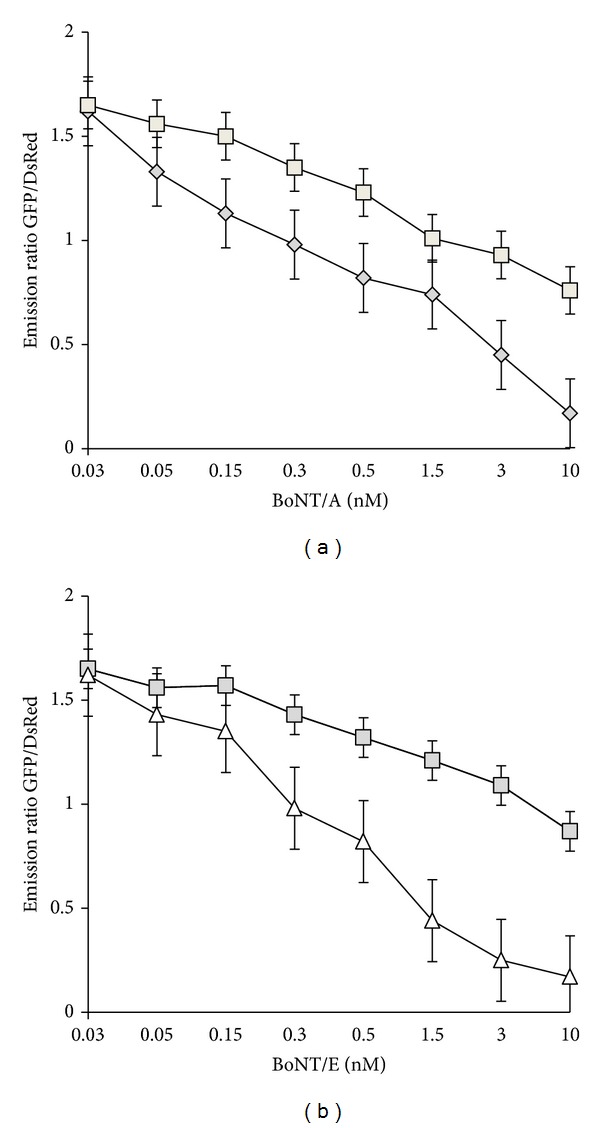
(a) BoNT/A complex activity and comparison with BoNT/A holotoxin. Clone 5A3 cells were subjected to assay protocol to test the activity of BoNT/A holotoxin and complex toxin. PC12 cells were grown in 96 well plates in the presence of indicated BoNT/A holotoxin (*⋄*) and complex toxin (□) at 0.03, 0.05, 0.15, 0.3, 1.5, 3.0, and 10 nM concentrations. Data are presented as means ± SD (*n* = 3). The fluorescence measurements were performed by BioTek microplate reader. (b) BoNT/E complex activity and comparison with BoNT/E holotoxin. Clone 5A3 cells were subjected to assay protocol to test the activity of BoNT/E holotoxin and complex toxin. PC12 cells were grown in 96 well plates in the presence of indicated BoNT/E holotoxin (△) and complex toxin (□) at 0.03, 0.05, 0.15, 0.3, 1.5, 3.0, and 10 nM concentrations. Data are presented as means ± SD (*n* = 3). The fluorescence measurements were performed by BioTek microplate reader.

In addition to holotoxin, we also checked the multiprotein complex form of the toxins BoNT/A and BoNT/E responses for the sensor assay. PC12 cells were grown in plates treated with 0.03–30 nM of BoNT/A and BoNT/E complex for different intervals (up to 72 hours) to show the toxin activity. There was only a moderate reduction in the FRET activity observed with a BoNT/A complex as compared to holotoxin treatments. At a higher concentration (30 nM) of BoNT/A treatment, there was a gradual reduction in the DsRed fluorescence indicating the action of toxin. This demonstrated that the BoNT/A and BoNT/E in their complex form are functionally less active in this detection method compared to holotoxin form of the toxin. The GFP and DsRed emissions were collected by fluorescence microplate reader (BioTek Instrument, Winooski, VT). Emissions were plotted as a function of BoNT/A holotoxin and complex as shown in Figure 5(a) and BoNT/E holotoxin and complex concentration as shown in Figure 5(b).

## 5. Conclusion

The cell-based assay developed here has the potential to be a rapid screening method to confirm the presence of functional botulinum toxin types A and E. The FRET-based reporter approach could be adapted to screen other BoNT serotypes with unique cleavage sites by using synthetic substrates labeled with different dyes. Cell-based reporters make it possible to gain further insights into toxin substrate recognition and cleavage in cells and to understand the BoNT cell biology. Here, we tested PC12 cell lines, which are large adherent cells, resist early apoptosis, and can be maintained easily with commercially available tissue culture medium and antibiotics. These stable clones could be produced in large quantities and are reproducible. In a single experiment, sufficient stable cells can easily be generated to populate twenty 96-well dishes. Alternatively, cells can also be cryopreserved with 50% viability upon thawing to provide lot-to-lot consistency. Even though the assay is of low sensitivity, it has the potential to be a high-throughput detection platform. We are currently exploring methods to increase the sensitivity of the assay and the available immuno-magnetic separation methods to counter the food matrix interference.
