# EXPRESSION OF CONCERN: Development of a Cell‐Based Functional Assay for the Detection of *Clostridium botulinum* Neurotoxin Types A and E

**DOI:** 10.1155/ijm/9821014

**Published:** 2026-03-26

**Authors:** International Journal of Microbiology

EXPRESSION OF CONCERN: U. Basavanna, T. Muruvanda, E. W. Brown, S. K. Sharma, “Development of a Cell‐Based Functional Assay for the Detection of *Clostridium botulinum* Neurotoxin Types A and E,” *International Journal of Microbiology*, 2013, 593219, https://doi.org/10.1155/2013/593219.

This Expression of Concern is for the above article, published online on 07 March 2013 in Wiley Online Library (https://wileyonlinelibrary.com), and has been issued by agreement between the journal Editor‐in‐Chief, Prof. Hidetoshi Urakawa; and John Wiley & Sons Ltd. The Expression of Concern has been agreed due to a concern raised on PubPeer [1] with Figure 1.

Specifically, Figure 1a appears similar to the image shown in Figure 1b of [2]. The authors neglected to cite the source of the image in the figure legend or in the references. They stated that this error was introduced during figure preparation, when the image from [2] was inserted as a placeholder. Due to the elapsed time since publication, the authors could not provide the original images or a replacement image of their own.

Therefore, the journal has decided to issue an Expression of Concern to inform and alert readers. The authors agree with this decision.
